# Eight new apterous *Lathrobium* species (Coleoptera, Staphylinidae) from Sichuan, Southwest China

**DOI:** 10.3897/zookeys.303.5328

**Published:** 2013-05-21

**Authors:** Zhong Peng, Li-Zhen Li, Mei-Jun Zhao

**Affiliations:** 1 Department of Biology, College of Life and Environmental Sciences, Shanghai Normal University, Shanghai, 200234, P. R. China

**Keywords:** Coleoptera, Staphylinidae, taxonomy, *Lathrobium*, new species, Sichuan, China

## Abstract

Eight apterous species of the paederine genus *Lathrobium* Gravenhorst, 1802 from the Chinese province Sichuan are described, illustrated, and distinguished from closely related and/or geographically close congeners: *Lathrobium erlangense* Peng & Li **sp. n.** (Erlang Shan), *Lathrobium blandum* Peng & Li **sp. n.** (Labahe N. R.), *Lathrobium yelense* Peng & Li **sp. n.** (Yele), *Lathrobium obscurum* Peng & Li **sp. n.** (Yele), *Lathrobium yinziweii* Peng & Li **sp. n.** (Yele), *Lathrobium illustre* Peng & Li **sp. n.** (Yele), *Lathrobium micangense* Peng & Li **sp. n.** (Micang Shan) and *Lathrobium agglutinatum* Assing & Peng **sp. n.** (Qingcheng Shan). The total number of described *Lathrobium* species from Sichuan now stands at 39, that of mainland China at 165.

## Introduction

So far, 157 species of the genus *Lathrobium* Gravenhorst have been reported from mainland China and the diversity is significantly greater than that of any other genus of the Paederinae. The provinces with the greatest diversity are Yunnan (58 species), followed by Sichuan (31 species), Shaanxi (20 species), and Zhejiang (17 species). However, these figures are still strongly biased. They do not reflect real diversities, but rather are a result of imbalanced collecting and study activity (Assing, in press a).

The topology of Sichuan is dominated by mountain regions (49.5%) and plateau (28.5%). The highest peak of Sichuan is the Gongga Shan at 7,556 m. East Sichuan is subject to the subtropical monsoon climate and the west is influenced by plateau alpine climate. Pine and beech forests form the main forest types in Sichuan (Yang 1988).

**Figure 1.**
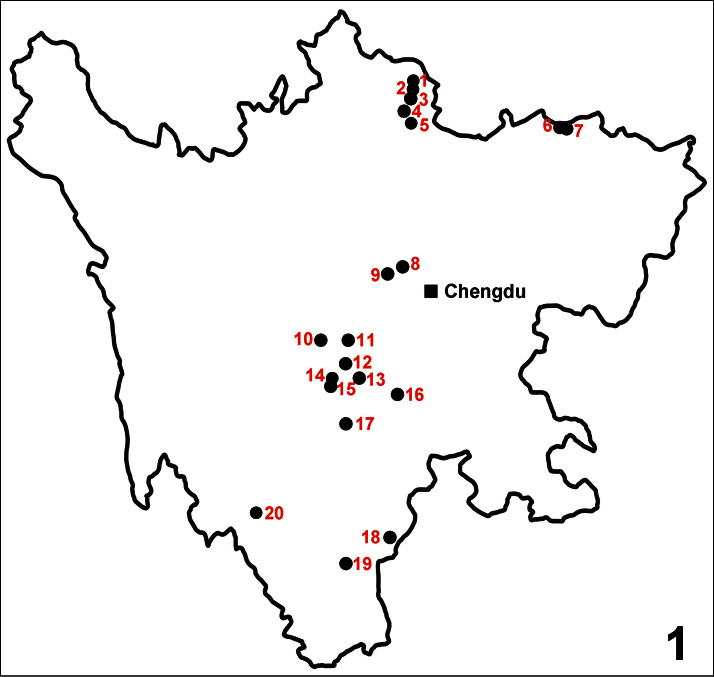
Distribution of the *Lathrobium* species in Sichuan: *Lathrobium biapicale* (**1–5**); *Lathrobium detruncatum* (**4**); *Lathrobium lentum* (**4**); *Lathrobium brevisternale* (**5**); *Lathrobium crassispinosum* (**6**); *Lathrobium sinense* (**6**); *Lathrobium longispinosum* (**6**); *Lathrobium serrilobatum* (**7**); *Lathrobium micangense* (**7**); *Lathrobium agglutinatum* (**8**); *Lathrobium bisuditum* (**9**); *Lathrobium verminatum* (**9**); *Lathrobium watanabei* (**10**); *Lathrobium acutissimum* (**11**); *Lathrobium labahense* (**11**); *Lathrobium blandum* (**11**); *Lathrobium aspinosum* (**12**); *Lathrobium bibaculatum* (**12**); *Lathrobium bispinigerum* (**12**); *Lathrobium erlangense* (**12**); *Lathrobium bihastatum* (**13**); *Lathrobium hailuogouense* (**14**); *Lathrobium celere* (**15**); *Lathrobium ventricosum* (**15**); *Lathrobium bisinuatum* (**16**); *Lathrobium conexum* (**16**); *Lathrobium coniunctum* (**16**); *Lathrobium ensigerum* (**16**); *Lathrobium hastatum* (**16**); *Lathrobium iunctum* (**16**); *Lathrobium yelense* (**17**); *Lathrobium yinziweii* (**17**); *Lathrobium obscurum* (**17**); *Lathrobium illustre* (**17**); *Lathrobium appendiculatum* (**18**); *Lathrobium bivirgatum* (**19**); *Lathrobium diffissum* (**19**); *Lathrobium hamulatum* (**19**); *Lathrobium formidabile* (**20**).

Schülke (2002) was the first to describe a micropterous *Lathrobium* species from Sichuan. Thirty additional species, most of them micropterous and locally endemic, were subsequently reported from this province by Peng et al. (2012), Assing et al. (2013) and Assing (in press b, c, d). In Sichuan, *Lathrobium* species have been described from the Emei Shan (6 species), the Gongga Shan (3 species), the Erlang Shan (3 species), the Labahe Nature Reserve (2 species), the Luoji Shan (3 species), the Xilingxue Shan (2 species), the region to the northwest of Kangding in the Daxue Shan (1 species), the Daxiang Ling (1 species), the Min Shan and adjacent mountain ranges in northern Sichuan (4 species), the Micang Shan at the border with Shaanxi (4 species), the region to the north of Jinyang in southern Sichuan (1 species), and the region to the northwest of Muli County in the Hengduan mountains (1 species). A map of the *Lathrobium* species from Sichuan Province is provided in Fig. 1.

In recent years, we surveyed the staphylinid fauna of several nature reserves in Sichuan Province (Erlang Shan; Labahe N. R.; Micang Shan; Qingcheng Shan and Yele), and collected numerous *Lathrobium* specimens. An examination of the material yielded eight undescribed apterous species, all remarkably different from the previously known species from China with respect to the male sexual characters.

## Material and methods

The following abbreviations are used in the text, with all measurements in millimeters:

**BL** length of body from the anterior margin of the labrum to the apex of the abdomen;

**FL** length of forebody from the anterior margin of the labrum to the posterior margin of the elytra;

**HL** from the anterior margin of the frons to the posterior margin of the head;

**HW** maximum width of head;

**PL** length of pronotum along midline;

**PW** maximum width of pronotum;

**EL** length of elytra from the apex of the scutellum to the posterior margin of the elytra;

**AL** length of the aedeagus from the apex of the ventral process to the base of the aedeagal capsule.

The type material is deposited in the Insect Collection of Shanghai Normal University, Shanghai, China (**SNUC**) and in the private collection of Volker Assing, Hannover (cAss).

## Taxonomy

### Lathrobium erlangense

Peng & Li sp. n.

urn:lsid:zoobank.org:act:3340FD66-09D4-48F5-8DAD-FC27B9492E84

http://species-id.net/wiki/Lathrobium_erlangense

Figs 2A , 3 , 13

#### Type material.

(1♂, 2♀♀). Holotype: ♂, labelled ‘CHINA: Sichuan Prov., Tianquan County Mt. Erlangshan, 29°52'N, 102°18'E, 13.vii.2012 alt. 2,200–2,300 m, Dai, Peng & Yin leg.’ (SNUC). Paratypes: 2 ♀♀, same label data as holotype (SNUC).

#### Description.

Measurements (in mm) and ratios: BL 5.84–8.06, FL 2.82–3.25, HL 0.83–0.92, HW 0.87–0.94, PL 1.17–1.26, PW 0.93–1.00, EL 0.56–0.67, AL 1.30, HL/HW 0.95–0.98, HW/PW 0.94, HL/PL 0.70–0.73, PL/PW 1.26, EL/PL 0.48–0.53.

**Figure 2.**
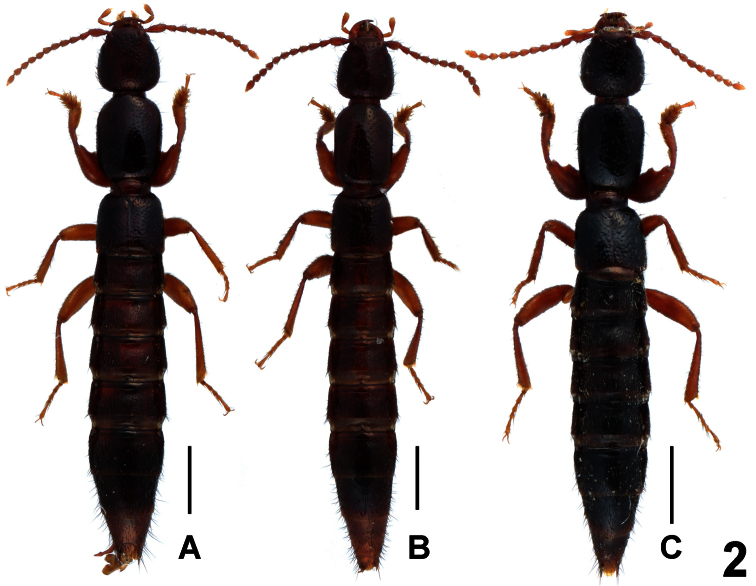
Habitus of *Lathrobium* spp., **A**﻿﻿﻿﻿ *Lathrobium erlangense* **B** *Lathrobium blandum* **C** *Lathrobium yelense*. Scale bars: 1.0 mm.

Habitus as in Fig. 2A. Body brown with paler apex, legs yellowish brown, antennae light brown.

Head subquadrate, distinctly dilated posteriorly; punctation coarse and of variable density, sparser in median dorsal area; interstices with fine microreticulation; eyes 1/4 times as long as postocular region in dorsal view.

Pronotum nearly parallel-sided; punctation somewhat sparser than that of head; impunctate midline broad; interstices without microsculpture.

Elytra approximately 0.48–0.53 times as long as pronotum; punctation fine, shallow, and moderately dense. Hind wings completely reduced. Protarsi with weakly pronounced sexual dimorphism.

**Figure 3.**
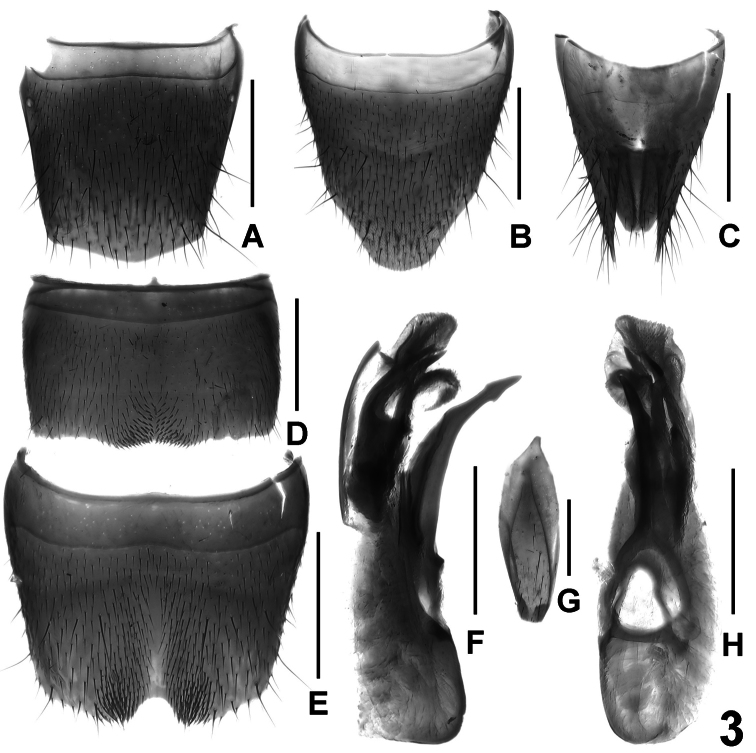
*Lathrobium erlangense*. **A** female tergite VIII **B** female sternite VIII **C** female tergites IX–X **D** male sternite VII **E** male sternite VIII **F** aedeagus in lateral view **G** male sternite IX **H** aedeagus in ventral view. Scale bars: 0.5 mm.

Abdomen with moderately fine and dense punctation, that of tergite VII noticeably sparser than that of anterior tergites; interstices with fine microsculpture; posterior margin of tergite VII without palisade fringe; tergite VIII without sexual dimorphism, convexly produced posteriorly (Fig. 3A).

Male. Sternites III-VI unmodified; sternite VII (Fig. 3D) transverse, symmetric, and with median impression of subtriangular shape posteriorly, this impression with cluster of distinctly modified, short and stout black setae, posterior margin weakly concave in the middle; sternite VIII (Fig. 3E) transverse, symmetric, and with shallow median impression, on either side of middle with cluster of weakly modified dark setae posteriorly, posterior excision small and of semi-circular shape; aedeagus (Figs 3F, 3H) with ventral process of distinctive shape, apical portion of dorsal plate long, lamellate and moderately sclerotized, basal portion of dorsal plate very short and weakly sclerotized, internal sac with distinctly sclerotized spines.

Female. Sternite VIII (Fig. 3B) much longer than tergite VIII, distinctly produced and finely pubescent posteriorly; tergite X (Fig. 3C) 1.1 times as long as the undivided antero-median portion of tergite IX (Fig. 3C).

#### Distribution and biological notes.

The species is known only from one locality in the Erlang Shan, Sichuan. The specimens were collected at an altitude of 2,200–2,300 m. The holotype was sifted from rhododendron leaves and soil on the east slope of a dry ditch in a rhododendron forest (Fig. 13).

#### Etymology.

The species is named after the mountain where the type locality locality is situated (“Erlang Shan”).

#### Comparative notes.

Based on the male and female sexual characters, *Lathrobium erlangense* undoubtedly belongs to the *Lathrobium bibaculatum* group (Assing, in press c). The similarly derived morphology of the aedeagus (somewhat spear-shaped ventral process, rather massive internal spines), as well as the similar modifications of the male sternites VII and VIII suggest that it is the adelphotaxon of *Lathrobium bibaculatum* Assing (in press c) from the Daxiang Ling, from which it is distinguished by somewhat smaller body size and by the more slender ventral process of the aedeagus.

### Lathrobium blandum

Peng & Li sp. n.

urn:lsid:zoobank.org:act:851DA1BD-04AD-4C4B-8258-C885CA682F6E

http://species-id.net/wiki/Lathrobium_blandum

Figs 2B , 4 , 14

#### Type material.

(1 ♂). Holotype: ♂, labelled ‘CHINA: Sichuan Prov., Tianquan County Labahe N. R., 30°10'N, 102°25'E, 12.vii.2012 alt. 2,200–2,300 m, Dai, Peng & Yin leg.’ (SNUC).

#### Description.

Measurements (in mm) and ratios: BL 8.62, FL 3.39, HL 0.98, HW 0.94, PL 1.31, PW 0.98, EL 0.70, AL 1.72, HL/HW 1.04, HW/PW 0.96, HL/PL 0.75, PL/PW 1.34, EL/PL 0.53.

Habitus as in Fig. 2B. Body light brown with paler apex, legs yellowish brown, antennae light brown.

Head weakly oblong; punctation moderately coarse and sparse, sparser in median dorsal portion; interstices with shallow microreticulation; eyes 1/5 times as long as postocular region in dorsal view.

Pronotum slender; punctation similar to that of head; impunctate midline moderately broad; interstices without microsculpture.

Elytra 0.53 times as long as pronotum; punctation shallow, moderately dense, and rather weakly defined. Hind wings completely reduced.

Abdomen with fine and dense punctation, that of tergite VII sparser than that of anterior tergites; interstices with fine microsculpture; posterior margin of tergite VII without palisade fringe.

**Figure 4.**
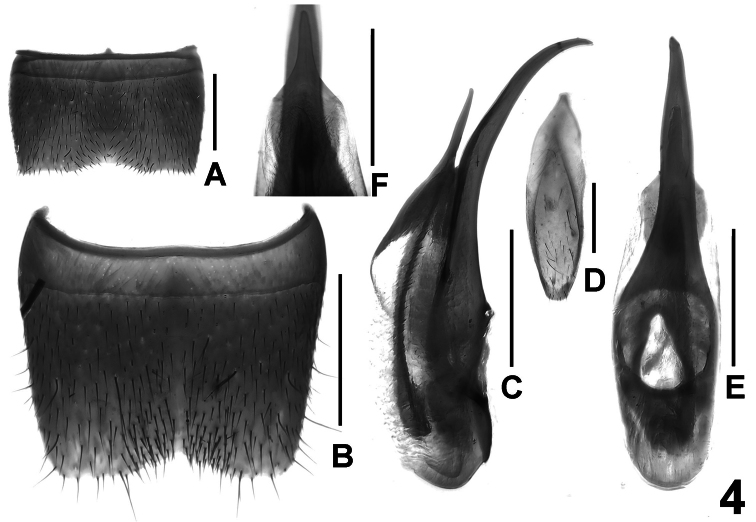
*Lathrobium blandum*. **A** male sternite VII **B** male sternite VIII **C** aedeagus in lateral view **D** male sternite IX **E** aedeagus in ventral view **F** apical portion of aedeagus in dorsal view. Scale bars: 0.5 mm.

Male. Sternites III-VI unmodified; sternite VII (Fig. 4A) transverse and with shallow postero-median impression, this impression with weakly modified setae, posterior margin concave in the middle; sternite VIII (Fig. 4B) transverse and impressed along the middle, on either side of this impression with short setae posteriorly, posterior margin broadly concave; sternite IX (Fig. 4D) nearly symmetric; aedeagus as in Figs 4C, 4E; ventral process evenly curved, slender, and apically acute in lateral view; dorsal plate (Fig. 4F) moderately sclerotized and with long apical portion, apically acute in dorsal view; basal portion short and thin; internal sac with long and slender sclerotized spine.

Female. Unknown.

#### Distribution and biological notes.

The species is known only from one locality in the Labahe Natural Reserve, Sichuan. The holotype was collected by sifting leaf litter and weeds from the floor of the hardwood forest with *Morus cathayana* and *Lonicera* on a westward slope at an altitude of 2,200–2,300 m (Fig. 14).

#### Etymology.

The specific epithet (Latin, adjective: seductive) alludes to the long and slender internal spine of the aedeagus.

#### Comparative notes.

The morphology of the aedeagus suggests that *Lathrobium blandum* belongs to the *Lathrobium curvatissimum* group (Assing, in press a), which previously included five species from Yunnan (Assing, in press a) and two species from Sichuan (Assing, in press b, c), with which the new species shares the elongated and curved ventral process, and the long apical portion of the dorsal plate of the aedeagus. It is distinguished from the other representatives of this group by the less slender head, the shape and chaetotaxy of the male sternites VIII, as well as by the long and slender sclerotized spine in the internal sac of aedeagus.

### Lathrobium yelense

Peng & Li sp. n.

urn:lsid:zoobank.org:act:3C6F3FB3-37B7-4289-8A1C-5A3D6A93AE7C

http://species-id.net/wiki/Lathrobium_yelense

Figs 2C , 5 , 15

#### Type material.

(4 ♂♂, 2 ♀♀). Holotype: ♂, labelled ‘CHINA: Sichuan Prov., Shimian County, Liziping. Yele, 28°54'N, 102°13'E, 15.vii.2012 alt. 2,600 m, Dai, Peng & Yin leg.’ (SNUC). Paratypes: 3 ♂♂, 2 ♀♀, same label data as holotype (SNUC).

#### Description.

Measurements (in mm) and ratios: BL 5.95–7.39, FL 2.83–3.20, HL 0.87–0.93, HW 0.88–0.92, PL 1.15–1.18, PW 0.92–0.96, EL 0.63–0.70, AL 1.24–1.29, HL/HW 0.99–1.01, HW/PW 0.94–0.96, HL/PL 0.76–0.79, PL/PW 1.23–1.25, EL/PL 0.55–0.59.

Habitus as in Fig. 2C. Body blackish brown with paler apex, legs and antennae brown to light brown.

Head subquadrate; punctation moderately coarse and rather sparse, slightly sparser in median dorsal portion; interstices with fine microreticulation; eyes 1/4 times as long as postocular region in dorsal view.

Pronotum nearly parallel-sided; punctation similar to that of head; impunctate midline moderately broad; interstices without microsculpture.

Elytra 0.55–0.59 times as long as pronotum; punctation moderately dense, shallow, and weakly defined. Hind wings completely reduced. Protarsi with moderately pronounced sexual dimorphism.

Abdomen with fine and dense punctation, that of tergite VII slightly sparser than that of anterior tergites; interstices with shallow microsculpture; posterior margin of tergite VII without palisade fringe; tergite VIII with moderately pronounced sexual dimorphism.

**Figure 5.**
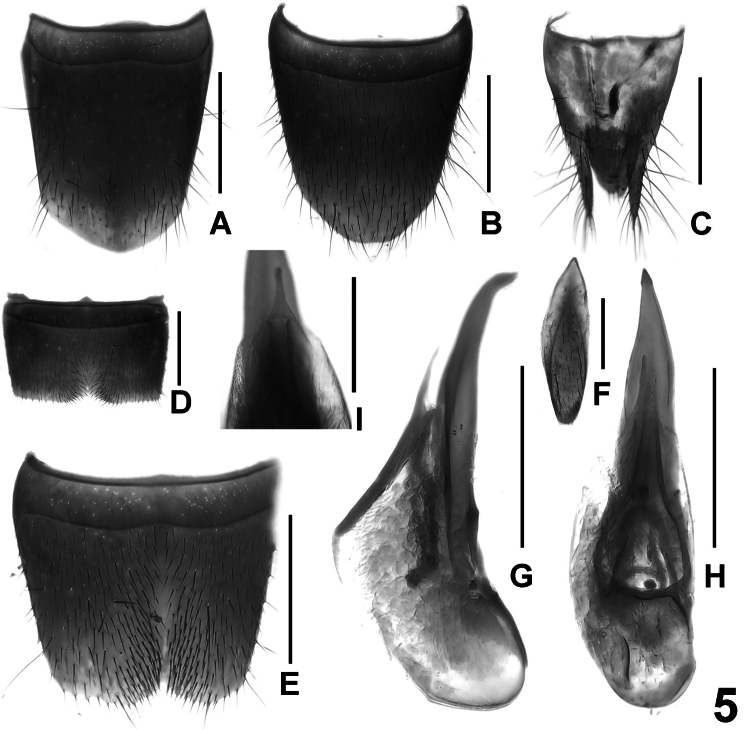
*Lathrobium yelense*. **A** female tergite VIII **B** female sternite VIII **C** female tergites IX–X. **D** male sternite VII **E** male sternite VIII **F** male sternite IX **G** aedeagus in lateral view **H** aedeagus in ventral view **I** apical portion of aedeagus in dorsal view. Scale bars: **A**–**H** 0.5 mm; **I** 0.25 mm.

Male. Tergite VIII with weakly convex posterior margin; sternites III-VI unmodified; sternite VII (Fig. 5D) transverse, with weakly modified setae in shallow postero-median impression, posterior margin concave in the middle; sternite VIII (Fig. 5E) moderately transverse and impressed along the middle, on either side of this impression with numerous short and dark setae posteriorly, posterior margin shallowly concave in the middle; sternite IX (Fig. 5F) nearly symmetric; aedeagus as in Figs 5G, 5H; ventral process long and apically curved in lateral view; dorsal plate (Fig. 5I) weakly sclerotized and with long apical portion, apically acute in dorsal view; basal portion very short and thin; internal sac without distinct dark membranous structures and apically with moderately sclerotized structure.

Female. Tergite VIII (Fig. 5A) asymmetrically produced posteriorly; sternite VIII (Fig. 5B) longer than tergite VIII, distinctly produced posteriorly, posteriorly finely pubescent; tergite X (Fig. 5C) 0.4 times as long as the undivided antero-median portion of tergite IX (Fig. 5C).

#### Distribution and biological notes.

This species is currently known only from the type locality. Some of the type specimens were collected by sifting bamboo leaves and humus from the floor of a bamboo forestat an altitude of 2,600 m (Fig. 15).

#### Etymology.

The species is named after its type locality: “Yele”.

#### Comparative notes.

Based on the male and female sexual characters, particularly the long and large dorsal plate, and the presence of an apical internal structure of the aedeagus, *Lathrobium yelense* may belong belongs to the *Lathrobium ensigerum* group (Assing et al. 2013). It is distinguished from the other representatives of this group by the sparser punctation on the head, the shape and chaetotaxy of the male sternite VIII, the oblong female tergite VIII, as well as by the morphology of the aedeagus.

### Lathrobium obscurum

Peng & Li sp. n.

urn:lsid:zoobank.org:act:930E1048-C5E3-4923-B2C8-53F5DB650872

http://species-id.net/wiki/Lathrobium_obscurum

Figs 6A , 7

#### Type material.

(1 ♂). Holotype: ♂, labelled ‘CHINA: Sichuan Prov., Shimian County, Liziping. Yele, 28°54'N, 102°13'E, 15.vii.2012 alt. 2,600 m, Dai, Peng & Yin leg.’ (SNUC).

#### Description.

Measurements (in mm) and ratios: BL 10.17, FL 4.10, HL 1.15, HW 1.20, PL 1.55, PW 1.20, EL 0.83, AL 2.38, HL/HW 0.96, HW/PW 1.00, HL/PL 0.74, PL/PW 1.29, EL/PL 0.54.

**Figure 6.**
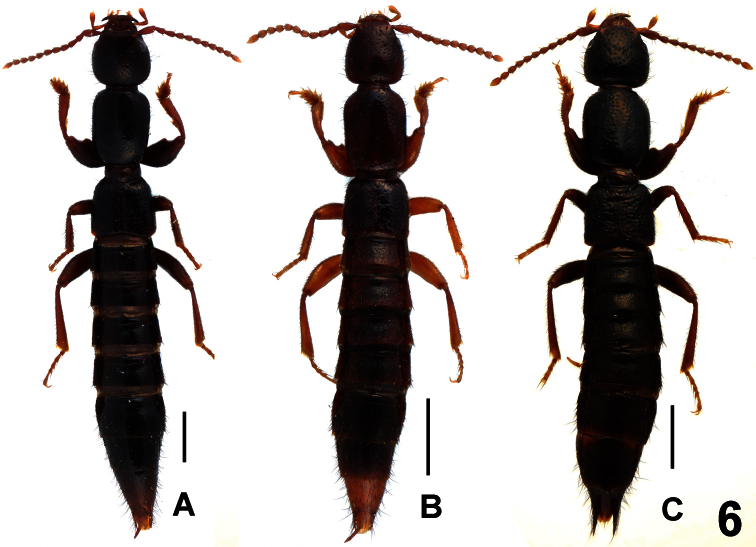
Habitus of *Lathrobium* spp., **A** *Lathrobium obscurum* **B** *Lathrobium yinziweii* **C** *Lathrobium illustre*. Scale bars: 1.0 mm.

Habitus as in Fig. 6A. Body blackish brown with paler apex, legs dark brown, antennae dark brown to brown.

Head weakly transverse; punctation coarse and moderately dense, sparser in median dorsal portion; interstices with fine microreticulation; eyes 1/4 times as long as postocular region in dorsal view.

Pronotum nearly parallel-sided; punctation somewhat sparser than that of head; impunctate midline broad; interstices without microsculpture.

Elytra 0.54 times as long as pronotum; punctation fine, shallow, and moderately dense. Hind wings completely reduced.

Abdomen with fine and dense punctation, that of tergite VII sparser than that of anterior tergites; interstices with shallow microsculpture; posterior margin of tergite VII without palisade fringe.

**Figure 7.**
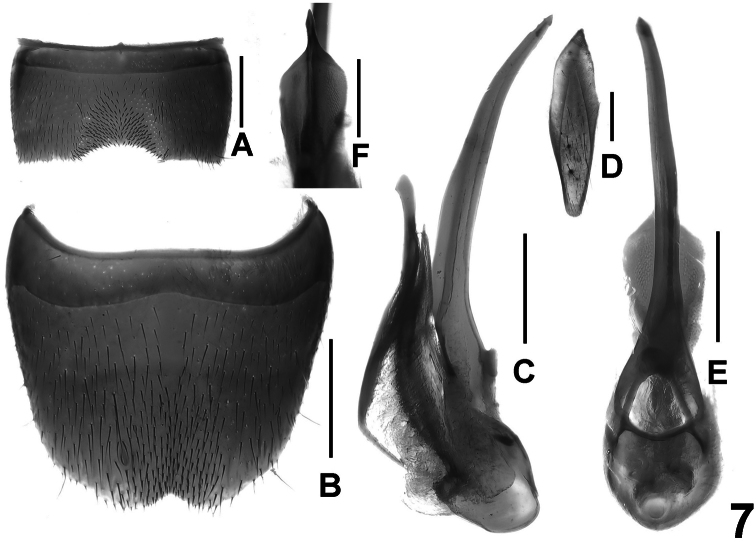
*Lathrobium obscurum*. **A** male sternite VII **B** male sternite VIII **C** aedeagus in lateral view **D** male sternite IX **E** aedeagus in ventral view **F** apical portion of aedeagus in dorsal view. Scale bars: 0.5 mm.

Male. Sternites III-VI unmodified; sternite VII (Fig. 7A) strongly transverse, with median impression of triangular shape posteriorly, this impression with numerous distinctly modified, short and stout black setae; posterior margin distinctly concave in the middle; sternite VIII (Fig. 7B) transverse and broadly impressed along the middle, this impression with short modified setae, posterior margin shallowly concave in the middle; sternite IX (Fig. 7D) nearly symmetric; aedeagus as in Figs 7C, 7E; ventral process long, slender and evenly curved; dorsal plate (Fig. 7F) sclerotized and with long apical portion, apically acute in dorsal view and weakly curved in lateral view; basal portion short and thin; internal sac without sclerotized spines and with membranous structures.

Female. Unknown.

#### Distribution and biological notes.

This species is currently known only from the type locality. The holotype was collected by sifting litter of bamboo and rhododendronfrom the floor of rhododendron forestat an altitude of 2,600 m.

#### Etymology.

The specific epithet (Latin, adjective: dark) alludes to the dark brown coloration of the legs.

#### Comparative notes.

The morphology of the aedeagus suggests that *Lathrobium obscurum* belongs to the *Lathrobium curvatissimum* group (Assing, in press a), which previously included five species from Yunnan (Assing, in press a) and two species from Sichuan (Assing, in press b, c), with which the new species shares the long, evenly curved ventral process, the long apical portion of the dorsal plate of the aedeagus and the absence of a distinct posterior excision of the male sternite VIII. It is distinguished from the other representatives of this group by the shape and chaetotaxy of the male sternites VII, as well as by with membranous structures in the internal sac of aedeagus.

### Lathrobium yinziweii

Peng and Li sp. n.

urn:lsid:zoobank.org:act:75FC14AD-DF7D-4EBE-95D2-18AC29FBBF1A

http://species-id.net/wiki/Lathrobium_yinziweii

Figs 6B , 8 , 15

#### Type material.

(3 ♂♂, 4 ♀♀). Holotype: ♂, labelled ‘CHINA: Sichuan Prov., Shimian County, Liziping. Yele, 28°54'N, 102°13'E, 15.vii.2012 alt. 2,600 m, Dai, Peng & Yin leg.’ (SNUC). Paratypes: 2 ♂♂, 4 ♀♀, same label data as holotype (SNUC).

#### Description.

Measurements and ratios: BL 5.50–6.89, FL 2.40–2.82, HL 0.72–0.79, HW 0.75–0.78, PL 0.96–1.07, PW 0.77–0.82, EL 0.51–0.57, AL 1.67–1.72, HL/HW 0.96–1.01, HW/PW 0.95–0.97, HL/PL 0.73–0.76, PL/PW 1.25–1.30, EL/PL 0.52–0.54.

Habitus as in Fig. 6B. Body light brown with paler apex, legs yellowish brown, antennae light brown.

Head subquadrate (HL/HW 0.96–1.01); punctation moderately coarse and sparse, sparser in median dorsal portion; interstices with fine microreticulation; eyes 1/5–1/4 times as long as postocular region in dorsal view.

Pronotum slender; punctation somewhat denser than that of head; impunctate midline broad; interstices without microreticulation.

Elytra 0.52–0.54 times as long as pronotum; punctation fine, shallow, and moderately dense. Hind wings completely reduced. Protarsi with moderately pronounced sexual dimorphism.

Abdomen with fine and dense punctation, that of tergite VII sparser than that of anterior tergites; interstices with shallow microsculpture; posterior margin of tergite VII without palisade fringe; tergite VIII with moderately pronounced sexual dimorphism.

**Figure 8.**
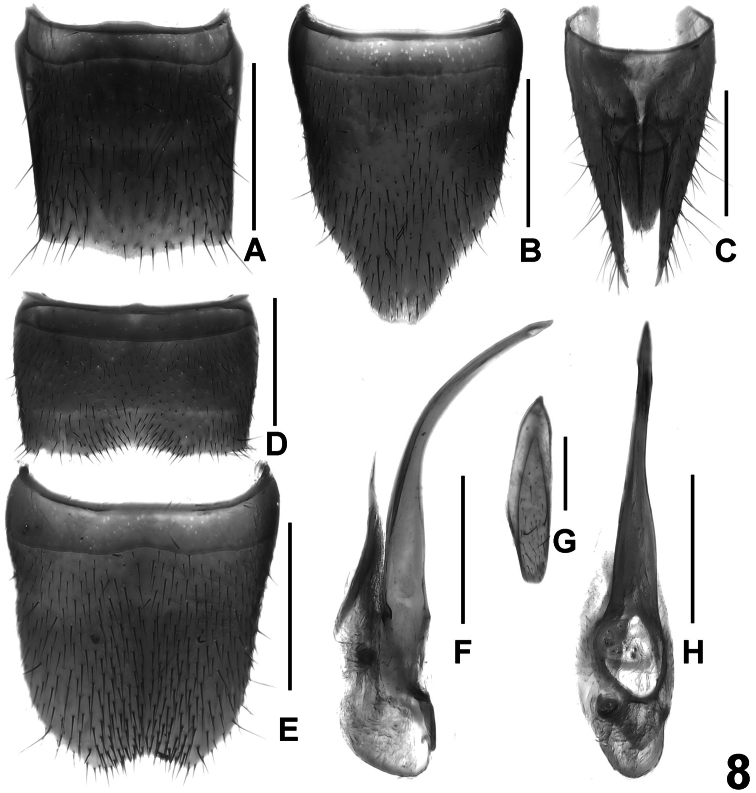
*Lathrobium yinziweii*. **A** female tergite VIII **B** female sternite VIII **C** female tergites IX–X. **D** male sternite VII **E** male sternite VIII **F** aedeagus in lateral view **G** male sternite IX **H** aedeagus in ventral view. Scale bars: 0.5 mm.

Male. Tergite VIII with nearly truncate posterior margin; sternites III-VI unmodified; sternite VII (Fig. 8D) transverse and with shallow postero-median impression, pubescence very weakly modified, posterior margin concave in the middle; sternite VIII (Fig. 8E) broadly impressed along the middle, this impression with short modified setae, posterior margin shallowly concave in the middle; sternite IX (Fig. 8G) asymmetric; aedeagus as in Figs 8F, 8H; ventral process very long, slender, evenly curved, and apically indistinctly spear-shaped; basal portion of dorsal plate very short; internal sac with membranous structures and usual ring-shaped structure.

Female. Posterior margin of tergite VIII (Fig. 8A) weakly convex; sternite VIII (Fig. 8B) much longer than tergite VIII and rather narrowly produced posteriorly; tergites IX-X (Fig. 8C) long and slender, tergite X (Fig. 8C) 1.4 times as long as antero-median portion of tergite IX (Fig. 8C).

#### Distribution and biological notes.

This species is currently known only from the type locality. One male was collected by sifting bamboo leaves and humus from the floor of the bamboo forest (Fig. 15). The other specimens were collected by sifting litter of bamboo and rhododendronfrom the floor of a rhododendron forestat an altitude of 2,600 m.

#### Etymology.

The species is named after Yin Zi-Wei, who collected the type specimens.

#### Comparative notes.

*Lathrobium yinziweii* is evidently closely related to *Lathrobium diffissum* (Assing, in press b) from the Luoji Shan. Both species share an aedeagus of similar morphology (ventral process long, slender, curved, and apically indistinctly spear-shaped; basal portion of dorsal plate very short; internal sac without sclerotized structures), a similar shape of the male sternite VII and sternite VIII (the posterior margin concave in the middle), a male sternite VIII with dense, but not distinctly modified pubescence, and a long and undivided antero-median portion of the female sternite IX. *Lathrobium yinziweii* is distinguished by the shape of the male sternite VIII (not transverse and with posterior excision), by the morphology of the aedeagus (ventral process evenly curved and slender; dorsal plate longer), by the shape of female sternite VIII, and by the long and slender female tergite IX-X.

### Lathrobium illustre

Peng & Li sp. n.

urn:lsid:zoobank.org:act:D980B711-7DFD-458C-8DA5-F1181F6F38FC

http://species-id.net/wiki/Lathrobium_illustre

Figs 6C , 9 , 15

#### Type material.

(1 ♂, 2 ♀♀). Holotype: ♂, labelled ‘CHINA: Sichuan Prov., Shimian County, Liziping. Yele, 28°54'N, 102°13'E, 15.vii.2012 alt. 2,600 m, Dai, Peng & Yin leg.’ (SNUC). Paratypes: 2 ♀♀, same label data as holotype (SNUC).

#### Description.

Measurements (in mm) and ratios: BL 7.84–9.23, FL 3.34–3.77, HL 1.02–1.05, HW 1.11–1.15, PL 1.44–1.50, PW 1.18–1.22, EL 0.74–0.78, AL 1.76, HL/HW 0.91–0.92, HW/PW 0.94, HL/PL 0.70–0.71, PL/PW 1.22–1.23, EL/PL 0.51–0.52.

Habitus as in Fig. 6A. Body dark brown with paler apex, legs and antennae dark brown to light brown.

Head weakly transverse; punctation coarse and moderately sparse, sparser in median dorsal portion; interstices with fine microreticulation; eyes 1/4–3/8 times as long as postocular region in dorsal view.

Pronotum weakly convex in dorsal view; punctation somewhat denser than that of head; impunctate midline broad; interstices without microreticulation and shining.

Elytra 0.51–0.52 times as long as pronotum; punctation fine, shallow, and moderately dense. Hind wings completely reduced. Protarsi with weakly pronounced sexual dimorphism.

**Figure 9.**
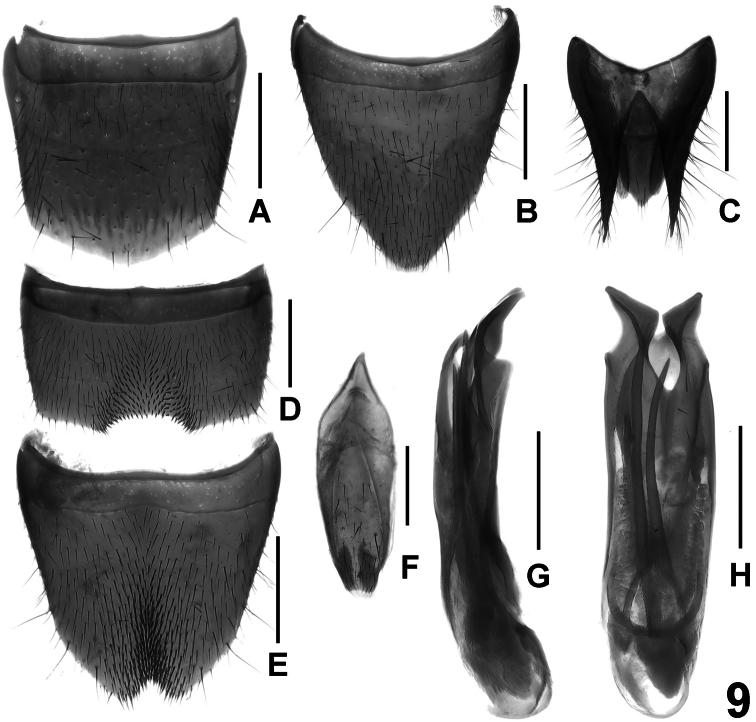
*Lathrobium illustre*. **A** female tergite VIII **B** female sternite VIII **C** female tergites IX–X. **D** male sternite VII **E** male sternite VIII **F** male sternite IX **G** aedeagus in lateral view **H** aedeagus in ventral view. Scale bars: 0.5 mm.

Abdomen with fine and dense punctation, that of tergite VII sparser than that of anterior tergites; interstices with shallow microsculpture; posterior margin of tergite VII without palisade fringe; tergite VIII without sexual dimorphism; posterior margin broadly convex (Fig. 9A).

Male. Sternites III-VI unmodified; sternite VII (Fig. 9D) transverse, with median impression of triangular shape posteriorly, this impression with numerous distinctly modified, short and stout black setae; posterior margin distinctly concave in the middle; sternite VIII (Fig. 9E) impressed along the middle, this impression with dense short setae, posterior excision small and nearly V-shaped; sternite IX (Fig. 9F) symmetric; aedeagus as in Figs 9G, 9H; ventral process bilobed apically, asymmetric and of distinctive shape; dorsal plate long and sclerotized; internal sac with two long and slender sclerotized spines.

Female. Sternite VIII (Fig. 9B) much longer than tergite VIII, distinctly produced posteriorly; tergite X (Fig. 9C) 5.7 times as long as antero-median portion of tergite IX (Fig. 9C).

#### Distribution and biological notes.

This species is currently known only from the type locality. The specimens were collected by sifting bamboo leaves and humus from the floor of a bamboo forestat an altitude of 2,600 m (Fig. 15).

#### Etymology.

The specific epithet (Latin, adjective: shining) alludes to the shining pronotum.

#### Comparative notes.

Based on the apically bilobed ventral process and the anteriorly short and undivided median portion of the female tergite IX, *Lathrobium illustre* may belong belongs to the *Lathrobium fissispinosum* group. It is distinguished from the other representatives of this group by the shining pronotum, the shape and chaetotaxy of the male sternite VII and male sternite VIII, as well as by the morphology of the aedeagus.

### Lathrobium micangense

Peng & Li sp. n.

urn:lsid:zoobank.org:act:EF416164-BE8D-437F-8DD7-ABDE4F4BB91A

http://species-id.net/wiki/Lathrobium_micangense

Figs 10A , 11 , 16

#### Type material.

(1 ♂). Holotype: ♂, labelled ‘CHINA: Sichuan Prov., Nanjiang County Mt. Micangshan, 32°39’N, 107°01’E, 27.iv.2008 alt. 1,800 m, Huang & Xu leg.’ (SNUC).

#### Description.

Measurements (in mm) and ratios: BL 6.78, FL 2.68, HL 0.83, HW 0.85, PL 1.11, PW 0.93, EL 0.56, AL 1.46, HL/HW 0.98, HW/PW 0.91, HL/PL 0.75, PL/PW 1.19, EL/PL 0.50.

**Figure 10.**
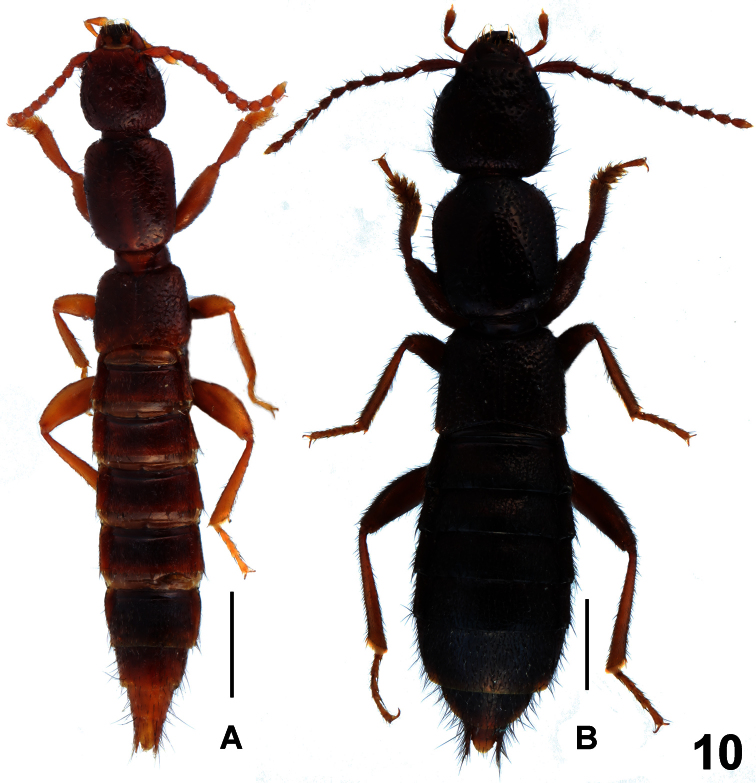
Habitus of *Lathrobium* spp., **A** *Lathrobium micangense* **B** *Lathrobium agglutinatum*. Scale bars: 1.0 mm.

Habitus as in Fig. 10A. Body reddish brown with paler apex, legs light brown, antennae reddish brown to yellowish brown.

Head subquadrate; punctation moderately coarse and sparse, sparser in median dorsal portion; interstices with fine microreticulation; eyes 1/4 times as long as postocular region in dorsal view.

Pronotum nearly parallel-sided; punctation somewhat denser than that of head; impunctate midline broad; interstices without microsculpture.

Elytra 0.50 times as long as pronotum; punctation moderately dense, defined or weakly defined. Hind wings completely reduced.

Abdomen with fine and dense punctation, that of tergite VII sparser than that of anterior tergites; interstices with shallow microsculpture; posterior margin of tergite VII without palisade fringe.

**Figure 11.**
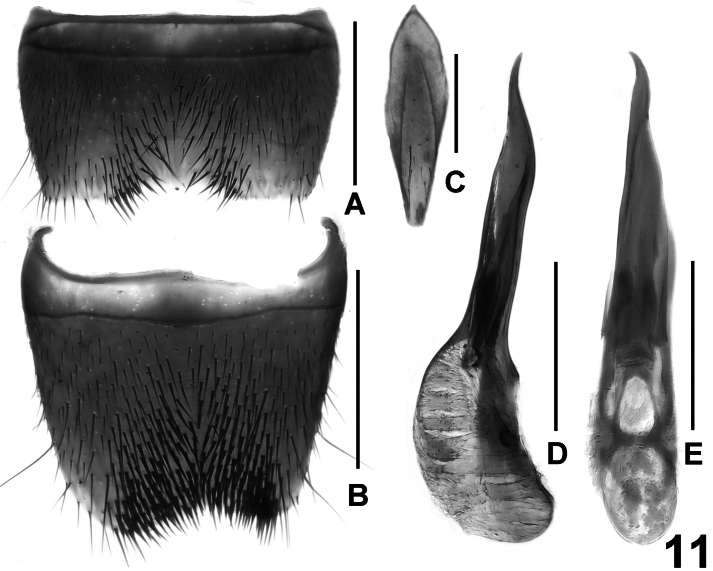
*Lathrobium micangense*. **A** male sternite VII **B** male sternite VIII **C** male sternite IX **D** aedeagus in lateral view **E** aedeagus in ventral view. Scale bars: 0.5 mm.

Male. Sternites III-VI unmodified; sternite VII (Fig. 11A) transverse and deeply impressed in postero-median portion, this impression with several long dark setae, posterior margin weakly concave in the middle; sternite VIII (Fig. 11B) distinctly asymmetric and broadly impressed in postero-median portion, this impression with dense dark long setae, posterior margin broadly concave; sternite IX (Fig. 11G) asymmetric; aedeagus as in Figs 11D, 11E; ventral process long and asymmetric in ventral view; dorsal plate long and thin; internal sac with straight moderately sclerotized spine.

Female. Unknown.

#### Distribution and biological notes.

The species is known only from one locality in Micang Shan, Sichuan. The holotype was collected by sifting dry leaf litter and moss on a southward slope with *Prunus* at an altitude of 1,800 m (Fig. 16).

#### Etymology.

The species is named after its type locality: “Micang Shan”.

#### Comparative notes.

Based on the morphology of the aedeagus (the shapes and chaetotaxy of the male sternites VII and VIII, presence of a moderately sclerotized spine in the internal sac, asymmetric ventral process), *Lathrobium micangense* belongs to the *Lathrobium fissispinosum* group. The morphology of the ventral process and the similar shape, the chaetotaxy of the male sternite VII and the asymmetric male sternite VIII suggest that it is closely related to *Lathrobium longispinosum*, from which *Lathrobium micangense* differs by the smaller body, the arrangement of the modified setae of the male sternite VIII, the shapes of the ventral process and the dorsal plate of the aedeagus, and the straight moderately sclerotized internal spine of the aedeagus (*Lathrobium longispinosum*: spine weakly curved).

### Lathrobium agglutinatum

Assing & Peng sp. n.

urn:lsid:zoobank.org:act:4DBEE816-245C-4921-828B-F807A5F32488

http://species-id.net/wiki/Lathrobium_agglutinatum

Figs 10B , 12 , 17

#### Type material.

(3 ♂♂). Holotype: ♂, labelled ‘CHINA: Sichuan Prov., Dujiangyan City, Mt. Qingchengshan, 30°57'N, 103°28'E, 30.vii.2012 alt. 1,700 m, Dai, Peng & Yin leg.’ (SNUC). Paratypes: 1 ♂, same label data as holotype (SNUC); 1 ♂, ‘China (Sichuan) 1999, Qingcheng-shan, (Umg. Heavenly Old Village) 1000-1300 m, 18./20.VI. Heinz leg.’ (cAss).

#### Description.

Measurements (in mm) and ratios: BL 7.78–9.51, FL 4.00–4.28, HL 1.25–1.28, HW 1.38–1.40, PL 1.63–1.68, PW 1.40–1.43, EL 0.81–0.83, AL 1.65–1.70, HL/HW 0.91, HW/PW 0.98–0.99, HL/PL 0.77, PL/PW 1.16–1.17, EL/PL 0.49–0.50.

Habitus as in Fig. 5A. Body dark brown with paler apex, legs and antennae brown to light brown.

Head subquadrate; punctation dense and coarse; interstices with fine microreticulation; eyes 0.3 times as long as postocular region in dorsal.

Pronotum with weakly convex lateral margins in dorsal view; punctation somewhat sparser than that of head; impunctate midline narrow; interstices without microsculpture.

Elytra 0.49–0.50 times as long as pronotum; punctation shallow and much denser than that of pronotum; interstices without distinct microsculpture. Hind wings reduced.

Abdomen much broader than elytra, with fine and dense punctation, that of tergite VII sparser than that of anterior tergites; interstices with shallow microsculpture; posterior margin of tergite VII without palisade.

**Figure 12.**
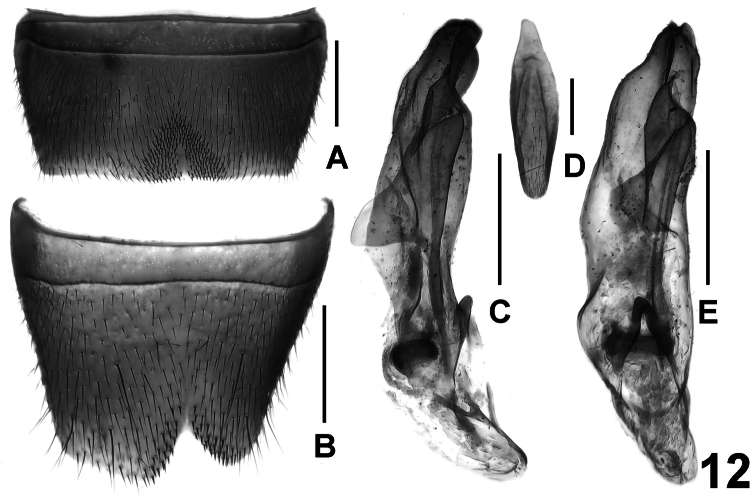
*Lathrobium agglutinatum*. **A** male sternite VII **B** male sternite VIII **C** aedeagus in lateral view **D** male sternite IX **E** aedeagus in ventral view. Scale bars: 0.5 mm.

Male. Sternites III-VI unmodified; sternite VII (Fig. 12A) strongly transverse and with short dark seta in triangular postero-median impression, posterior margin nearly truncate; sternite VIII (Fig. 12B) transverse and weakly impressed in postero-median portion, posterior excision pronounced, deep and asymmetric, anterior margin of this excavation with short dark setae; sternite IX (Fig. 12D) asymmetric; aedeagus as in Figs 12C, 12E; ventral process and dorsal plate fused; basal portion of aedeagus small; internal sac with usual ring-shaped structure.

Female. Unknown.

#### Distribution and biological notes.

The species is known only from one locality in the Qingcheng Shan, Sichuan. Two specimens were collected by sifting leaf litter and humus from the floor of a hardwood forest with Cherokee rose and *Rubus* at an altitude of 1,700 m (Fig. 17).

**Figures 13–17.**
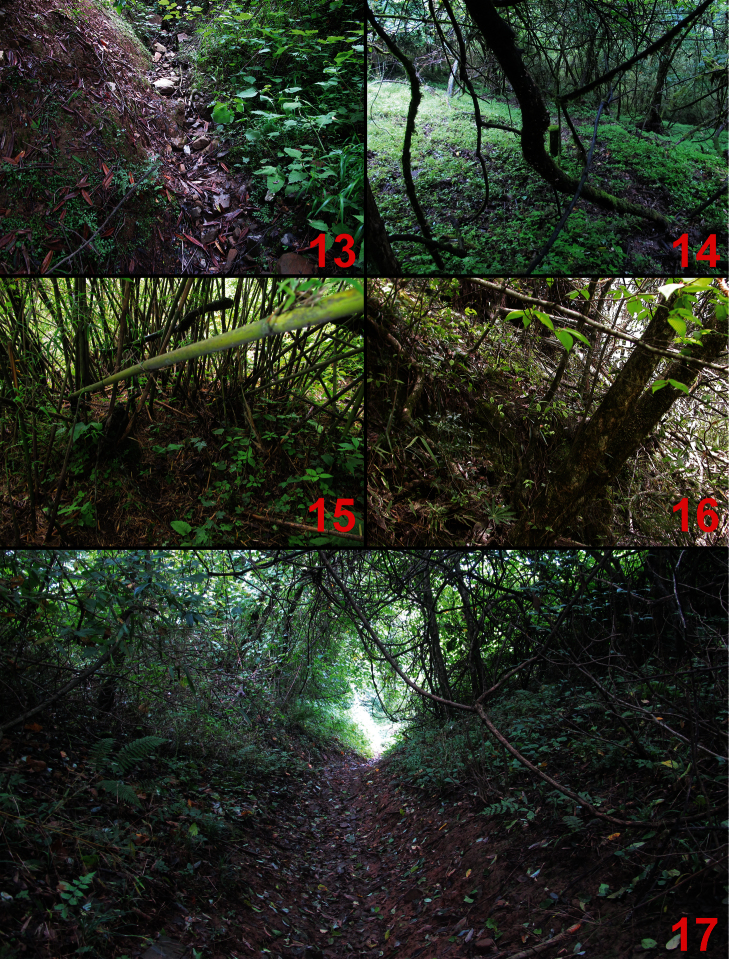
Habitats of the new species. **13** Erlang Shan, alt. 2,200–2,300 m (*Lathrobium erlangense* sp. n.) **14** Labahe Natural Reserve, alt. 2,200–2,300 m (*Lathrobium blandum* sp. n.) **15** Yele, alt. 2,600 m (*Lathrobium illustre* sp. n., *Lathrobium yinziweii* sp. n. and *Lathrobium yelense* sp. n.) **16** Micang Shan, alt. 1,800 m (*Lathrobium micangense* sp. n.) **17** Qingcheng Shan, alt. 1,700 m (*Lathrobium agglutinatum* sp. n.).

#### Etymology.

The specific epithet is the past participle of the Latin verb agglutinare (to glue together) and alludes to the fused ventral process and dorsal plate of the aedeagus.

#### Comparative notes.

*Lathrobium agglutinatum* is undoubtedly closely related to *Lathrobium conexum* and belongs to the *Lathrobium iunctum* group (Assing et al., 2013). This conclusion is supported by the similarly derived structure of the aedeagus (ventral process and dorsal plate fused, asymmetric, and slender; basal portion small; internal sac with small and weakly sclerotized basal sclerite); the similarly derived shape and chaetotaxy of the male sternite VIII (posterior excision asymmetric, the anterior margin of this excavation with short dark setae), and by the extremely similar external characters. Both species are best distinguished by the completely different shape and chaetotaxy of the male sternite VII and by the differently shaped apex of the aedeagus. For illustrations of the species of the *Lathrobium iunctum* group from the Emei Shan see Assing et al. (2013).

## Supplementary Material

XML Treatment for Lathrobium erlangense

XML Treatment for Lathrobium blandum

XML Treatment for Lathrobium yelense

XML Treatment for Lathrobium obscurum

XML Treatment for Lathrobium yinziweii

XML Treatment for Lathrobium illustre

XML Treatment for Lathrobium micangense

XML Treatment for Lathrobium agglutinatum
